# Positive Thyroid Peroxidase Antibody among Women with Polycystic Ovarian Syndrome Visiting an Infertility Clinic at a Tertiary Care Centre

**DOI:** 10.31729/jnma.8366

**Published:** 2023-12-31

**Authors:** Vijay Kumar Sharma, Sujata Baidya, Pratibha Kandel, Smrity Rajkarnikar, Apeksha Niraula, Raju Kumar Dubey, Mithileshwer Raut, Aseem Bhattarai, Eans Tara Tuladhar, Poonam Koirala

**Affiliations:** 1 Department of Clinical Biochemistry, Institute of Medicine, Tribhuvan University Teaching Hospital, Maharajgunj, Kathmandu, Nepal; 2 Department of Obstetrics and Gynaecology, Tribhuvan University Teaching Hospital, Maharajgunj, Kathmandu, Nepal

**Keywords:** *anti-thyroid autoantibodies*, *autoimmunity*, *infertility*, *prevalence*, *polycystic ovary syndrome*

## Abstract

**Introduction:**

Polycystic ovarian syndrome is the most common endocrine-metabolic disorder, affecting women of reproductive age groups, which shares various symptoms with thyroid dysfunctions. Despite it predisposition of aforesaid cohorts to autoimmunity, these etiologies have not adequately been studied in them. This study aimed to find out the prevalence of positive thyroid peroxidase antibodies among women with polycystic ovarian syndrome visiting an infertility clinic at a tertiary care centre.

**Methods:**

This was a cross-sectional study conducted at a tertiary care centre among patients visiting the infertility clinic at the Department of Obstetrics and Gynaecology from 21 September 2022 to 21 February 2023. Biochemical analysis of thyroid hormones, gonadal hormones, anti-mullerian hormone and thyroid peroxidase antibody were done in Abbott ARCHITECT ci4100 and SNIBE Maglumi 800 autoanalyzer. A convenience sampling method was used. The point estimate was calculated at a 95% Confidence Interval.

**Results:**

Among 70 participants, thyroid peroxidase antibody was positive in 16 (22.86%) (13.02-32.69, 95% Confidence Interval). The mean age of the patients was 28.25±5.26 years. In the individuals with thyroid-stimulating hormone below 2.5 mIU/l, 5 (31.25%) had positive thyroid peroxidase antibody titre.

**Conclusions:**

The prevalence of positive thyroid peroxidase antibodies among women with polycystic ovarian syndrome is similar to other studies done in similar settings. Regular monitoring of thyroid peroxidase antibodies is recommended in these women to guide conception in order to evade inevitable adverse pregnancy outcomes.

## INTRODUCTION

Polycystic ovarian syndrome (PCOS) is a common cause of infertility in women characterized by hyperandrogenism, polycystic ovarian morphology, ovarian dysfunction and hyperinsulinemia.^1^ It affects nearly 6-20% of women of the reproductive age group and 90-95% of anovulatory women seeking infertility treatment.^1,2^

PCOS shares symptoms of dyslipidemia, abnormal glucose metabolism, hypertension and insulin resistance with hypothyroidism, hence the association of thyroid disease and PCOS is a burgeoning area of research.^3^ The skein of biochemical alteration in PCOS and autoimmune thyroid disorders (AITD) crosstalk at various points.^4^ AITD is the most common autoimmunity associated with PCOS with an estimated prevalence of 5%.^5^ Despite the impending threat of grave cardiovascular outcomes and autoimmunity in PCOS, management of these individuals is mostly focused on current issues of infertility with thyroid autoimmunity in shade.

The primary objective was to find out the prevalence of positive thyroid peroxidase antibodies among women with polycystic ovarian syndrome visiting an infertility clinic at a tertiary care centre.

## METHODS

This is a descriptive cross-sectional study conducted among the patients visiting the infertility clinic from 21 September 2022 to 21 February 2023 at the Department of Obstetrics and Gynaecology, Tribhuvan University Teaching Hospital, Maharajgunj, Kathmandu, Nepal. The ethical approval was obtained from the Institutional Review Committee of the Institute of Medicine, Maharajgunj, Kathmandu [Reference number: 175(6-11 )E2079/080]. Written informed consent was taken from all the participants and/or guardians involved (in the case of minors). The clinical data were anonymized and de-identified to ensure confidentiality. Patients presenting with PCOS-like features diagnosed by a gynaecologist via clinical examination and pelvic ultrasonography (USG) based on Rotterdam criteria were enrolled.^6^ Patients with Cushing's syndrome, non-classical adrenal hyperplasia, other drug-induced androgen excess and those unwilling to participate in the study were excluded. The convenience sampling method was used. The sample size was calculated by using the following formula:


$$\begin{array}{ll} \text{n} & ={\text{Z}}^{2}\times \frac{\text{p}\times \text{q}}{{\text{e}}^{\text{2}}}\\ & =1.96^{2}\times \frac{0.082\times 0.918}{0.07^{2}}\\ & =60 \end{array}$$

Where,

n = minimum required sample size
Z = 1.96 at 95 % Confidence Interval (CI)
p = prevalence taken from the previous study, 8.2%^7^
q = 1-p
e = margin of error, 7%

The minimum sample size calculated was 60. However, we enrolled 70 patients diagnosed with PCOS.

The clinico-demographic data were collected at the sample site using a self-designed structured proforma and the blood samples were collected on the 2nd day of the menstrual cycle. All the samples were processed in the laboratory of the Department of Clinical Biochemistry. Biochemical analysis of hormonal parameters (free triiodothyronine (T3), free thyroxine (T4), thyroid-stimulating hormone (TSH), luteinizing hormone (LH), follicle-stimulating hormone (FSH), testosterone, estradiol) was done using Abbott ARCHITECT ci4100 Integrated System. Serum level of thyroid peroxidase antibody (anti-TPO) and anti-mullerian hormone (AMH) was measured using SNIBE Maglumi 800. The positive anti-TPO was defined as an elevated anti-TPO titre above 30 IU/ml.^8^

After entering the data in Microsoft Excel 2015, statistical analysis was performed using IBM SPSS Statistics version 22.0. The point estimate was calculated at a 95% CI.

## RESULTS

Among 70 PCOS-afflicted women, the prevalence of positive thyroid peroxidase antibody was evident in 16 (22.86%) (13.02-32.69, 95% CI). The study participants with positive anti-TPO titre ranged between the age groups of 21 and 40 years with a mean age of 8.25±5.26 years. The median value of the positive anti-TPO titre was 211.92 IU/ml (Table 1).

**Table 1:** Descriptive statistics of anthropometric and biochemical parameters in PCOS population with positive anti-TPO titre (n= 16).

Variable	Mean±SD	Median (Q1, Q3)
Age (years)	28.25±5.26	
BMI (kg/m^2^)	25.91±3.41	
Free T3 (pmol/L)	4.57±1.30	
Free T4 (pmol/L)	-	14.12 (12.48,14.5)
TSH (mlU/L)	-	3.80 (2.07, 4.80)
LH (IU/ml)	-	4.40 (2.60, 5.97)
FSH (IU/ml)	3.17±1.01	
Estradiol (pg/ml)	-	51.10 (36.88, 54.20)
Testosterone (ng/dl)	44.49±9.61	
LH/FSH ratio	1.56±0.69	
AMH (ng/ml)		5.03 (3.23, 8.78)
Anti-TPO (IU/ml)		211.92 (33.09, 708.40)

Despite having TSH levels below 2.5 mIU/l, a considerate proportion of individuals up to 5 (31.3%) had positive anti-TPO titre (Table 2).

**Table 2:** Status of different TSH ranges in anti-TPO positive cases (n= 16).

TSH Range (mIU/L)	n (%)
<2.5	5 (31.25)
2.5-4.5	6 (37.50)
4.5-10	4 (25)
>10	1 (6.25)

## DISCUSSION

In this study, 22.86% had positive anti-TPO titre among women with PCOS and most of the study population befitted the active reproductive age group. It complies with the fact that we enrolled only the individuals visiting the infertility clinic at TUTH to address their infertility issues.^9^ This was comparable to that reported in a previous study in which, TPO antibody was positive in 21.9% of the PCOS population. The finding coincides with that of a previous study in which anti-TPO was positive in 25% of PCOS patients.^10^ Similarly, other studies report the presence of positive anti-TPO in 19.6% and 26.9% of the PCOS cohorts, respectively.^11,12^ On the other hand, it was higher than the rate (8.2%) reported by in Vietnamese population.^7^ However, the threshold for positive and negative titre slightly differed in our study i.e. 30 IU/ml compared to the previous study^7^ i.e. 34 IU/ml and drastically as compared to the study^10^ i.e. 60 IU/ml.

Notably, anti-TPO was positive even in the individuals with the TSH values within the reference ranges. It infers that, in PCOS-afflicted women, the incidence of thyroid autoimmunity may endure even without overt thyroid dysfunctions. Higher autoantibody levels in these patients might as well be the culprit behind insufficient response to infertility treatment.^13,14^ Mounting evidence suggests that inflammation and autoimmunity can cause PCOS.^12^ Likewise, a higher prevalence of autoimmunity with PCOS might cause an increased risk of metabolic syndrome, infertility issues and adverse pregnancy outcomes.^15^

However, like many other studies, our study is confined to the PCOS status in the reproductive age group. Nevertheless, most women with PCOS are diagnosed in the child-bearing age, hence to mitigate adverse pregnancy outcomes, maintaining normal thyroid status is recommended.

## CONCLUSIONS

The prevalence of positive thyroid peroxidase antibodies among women with polycystic ovarian syndrome is similar to other studies done in similar settings.
